# *Vibrio cholerae* Gut Colonization of Zebrafish Larvae Induces a Dampened Sensorimotor Response

**DOI:** 10.3390/biomedicines13010226

**Published:** 2025-01-17

**Authors:** Isabella Cubillejo, Kevin R. Theis, Jonathan Panzer, Xixia Luo, Shreya Banerjee, Ryan Thummel, Jeffrey H. Withey

**Affiliations:** 1 Department of Biochemistry, Microbiology, and Immunology, Wayne State University, Detroit, MI 48201, USA; isabellacubillejo@wayne.edu (I.C.);; 2 Department of Ophthalmology, Visual and Anatomical Sciences, Wayne State University School of Medicine, Detroit, MI 48201, USA

**Keywords:** *Vibrio cholerae*, cholera, zebrafish larvae, microbiome, behavior

## Abstract

**Background:** Cholera is a diarrheal disease prevalent in populations without access to clean water. Cholera is caused by *Vibrio cholerae,* which colonizes the upper small intestine in humans once ingested. A growing number of studies suggest that the gut microbiome composition modulates animal behavior. Zebrafish are an established cholera model that can maintain a complex, mature gut microbiome during infection. Larval zebrafish, which have immature gut microbiomes, provide the advantage of high-throughput analyses for established behavioral models. **Methods:** We identified the effects of *V. cholerae* O1 El Tor C6706 colonization at 5 days post-fertilization (dpf) on larval zebrafish behavior by tracking startle responses at 10 dpf. We also characterized the larval gut microbiome using 16S rRNA sequencing. *V. cholerae*-infected or uninfected control groups were exposed to either an alternating light/dark stimuli or a single-tap stimulus, and average distance and velocity were tracked. **Results**: While there was no significant difference in the light/dark trial, we report a significant decrease in distance moved for C6706-colonized larvae during the single-tap trial. **Conclusion:** This suggests that early *V. cholerae* colonization of the larval gut microbiome has a dampening effect on sensorimotor function, supporting the idea of a link between the gut microbiome and behavior.

## 1. Introduction

Cholera is a profuse diarrheal disease spread via the fecal-oral route through contaminated food and water and is endemic in communities around the world without potable water and sanitary facilities [1]. Cholera patients pass a characteristic “rice-water stool” and, without rehydration treatment, are at risk of severe dehydration and death [2]. The World Health Organization estimates an annual 1–4 million cholera cases and upwards of 100,000 deaths internationally, with the most vulnerable demographic being children younger than 5 years old [3,4]. The global risk of cholera is characterized as very high, due to the increasing number of outbreaks and lack of vaccine availability. Prevalence is expected to worsen with climate change [5,6].

*Vibrio cholerae*, the etiological agent, is a motile Gram-negative bacillus found in aquatic environments [2,7]. *V. cholerae* isolates are classified into over 200 serogroups based on the somatic O-antigen. Some serogroups may cause mild to severe diarrheal symptoms, but only serogroups O1 and O139 cause cholera and cholera pandemics [1,7]. The O1 serogroup is further divided into two biotypes: classical and El Tor. The toxin-coregulated pilus (TCP) and cholera toxin (CT) are the two major virulence factors unique to these biotypes. These allow *V. cholerae* to colonize the upper small intestinal epithelium in humans and induce severe diarrheal symptoms, respectively [1,8,9,10,11]. Classical strains were the cause of the first six cholera pandemics beginning in 1817. However, beginning in 1961, El Tor became the dominant, if not primary, cause of the current seventh pandemic [12]. In comparison to El Tor strains, classical strains cause more severe diarrheal symptoms. However, El Tor strains are thought to be more persistent in colonization, as infection duration lasts longer [8,13]. Currently, some circulating El Tor strains, known as atypical or variant El Tor, have acquired some of the classical biotype virulence traits and cause more severe symptoms [14,15,16].

The human gut microbiome contains the majority of commensal bacteria in the body, which are crucial for digestion, nutrient absorption, metabolism, and the function of the immune system [17,18]. Firmicutes and Bacteroidetes are normally the dominant phyla of the human gut microbiome, though individual variation occurs due to genetics, age, sex, diet, and lifestyle, among other factors [19,20,21]. The mucus layer of the gut epithelium is essential to maintain a diverse microbiome, as it provides a surface for the resident gut microbiota and contains proteins for the bacteria to metabolize [22,23,24]. During cholera infection, *V. cholerae* colonizes the upper small intestine [10,25]. Here, CT induces physical efflux of ions and water, and the mucus shedding in tandem drastically decreases gut microbiome diversity. At this stage, *V. cholerae* are the most abundant bacteria in the now-altered human gut, and resident gut microbiota fluctuate in response to colonization [26,27].

Growing evidence suggests that dysbiosis (a significant change in the microbiome composition) of the gut is implicated in neurological conditions such as anxiety, depression, autism, and Parkinson’s [28,29,30]. Additionally, the presence of a microbiome is necessary for normal neurological development [31,32]. Because *V. cholerae* colonizes and perturbs the gut microbiome, it may be possible that infection affects neurological activity in a similar manner.

Current mammalian animal models used to study *V. cholerae* are useful for understanding pathogenesis, but require invasive procedures, use of infant animals, or antibiotic-treated adult animals to enable *V. cholerae* to colonize the gut [33,34,35,36,37,38]. Larval and adult zebrafish have been established as natural host models for *V. cholerae*. Infection via immersion is possible with pathogenic or non-O1 strains. Zebrafish and *V. cholerae* both originate from the Indian subcontinent, and zebrafish exhibit diarrheal symptoms similar to humans, though gut colonization does not require the same virulence factors required for humans [39,40,41]. A major advantage of the zebrafish model is that the entire infectious cycle, including colonization, competition with the intestinal microbiota, immune responses to infection, and transmission, can be studied, as fish are natural *V. cholerae* hosts. This infectious cycle closely parallels the human infectious cycle, but mammalian animal models cannot be used to study important components of the cycle, including competition with the intact gut microbiota. The zebrafish gut microbiome shifts over time among individuals and, without the use of antibiotics or invasive procedures required of other animal models, zebrafish have a mature gut microbiome before and during *V. cholerae* colonization [39,42,43,44]. Adult zebrafish will clear the *V. cholerae* infection on their own, and the duration varies based on the biotype strain used: classical strains are cleared within 72 h post-infection (hpi) while El Tor strains can colonize beyond 144 hpi [39]. Unpublished data from our lab suggest that El Tor persists for up to two weeks. Zebrafish larvae can also be infected via immersion at 5 days post-fertilization (dpf) when the gut opens and uptake of exogenous food begins [39,45]. The larval gut microbiome is primarily colonized by bacteria of the phylum Pseudomonadota and an unclassified Comamonadaceae [42,46].

Previous studies have found that microbial colonization is required in germ-free zebrafish larvae for normal swim behavior and neurobehavioral development [47,48]. One study found that axenic zebrafish larvae infected with *V. cholerae* at 1 dpf exhibited a decrease in hypoactivity at 10 dpf, while conventionally colonized zebrafish larvae infected with *V. cholerae* had no change in locomotor activity [48]. However, this was tested with a dark/light paradigm, whereas a second assay testing for a separate stimulus response would be beneficial to examining gut microbiome crosstalk with different regions of the larval brain and nervous system. An alternating light/dark assay tests the visual system, and a single-tap trial would test locomotor function separate from the visual system. Additionally, specificity of infection with a *V. cholerae* El Tor strain illuminates the role and effects of its long-term gut colonization on behavior and vice versa. Here, we report that *V. cholerae* El Tor C6706 colonization of the larval zebrafish gut has no effect on the response to a visual stimulus but does have a dampening effect on the response to an acoustic stimulus.

## 2. Materials and Methods

### 2.1. Zebrafish Husbandry

Wild-type AB larvae were used, and larvae were fasted at least 12 h before behavioral trials. During experimentation, larvae were placed in autoclaved water from the Aquaneering aquatic housing system filtered by reverse osmosis and maintained at pH 7.0 to 7.5. Tank water was conditioned with Instant Ocean salt (Aquarium Systems, Mentor, OH, USA) to a conductivity of 600 to 700 μS. Fish were kept in a glass-front incubator at 28 °C on a timed 14 h light, 10 h dark cycle. All animal protocols were approved by the Wayne State University IACUC.

### 2.2. Vibrio cholerae Infection

*V. cholerae* infection of zebrafish has been previously described [39,40]. Briefly, El Tor C6706 was incubated with aeration in 30 mL of Luria Broth (LB) agar with 100 µg/mL streptomycin at 37 °C for 12–14 h. Cells were centrifuged at 8000× *g* then washed and resuspended with 1× phosphate buffer saline (PBS) to a concentration of 10^9^ CFU/mL by measuring at OD600 nm. Serial dilutions were plated onto LB agar with 100 µg/mL streptomycin and 100 µg/mL X-galactosidase (X-gal) for confirmation. In 6-well plates (Fisher Scientific, Pittsburgh, PA, USA), the *V. cholerae* dilution was pipetted into 5 mL of autoclaved water from the Aquaneering system to an average concentration for both behavioral trials, each repeated in triplicate, of 1.16 × 10^7^ CFU per well. For the uninfected group, in separate 6-well plates, the same amount of sterile 1× PBS was used.

Due to variable larval counts available during experimentation, approximately 10–15 larvae at 5 dpf were then placed in each well with the inoculum (uninfected n = 88, C6706 n = 111). After 6 hpi, all larvae from both groups were removed from wells and separately placed in new wells with 5 mL of new autoclaved water. This rinsing process was repeated three times to wash off the initial inoculum as thoroughly as possible. The larvae were then placed in beakers with 80 mL of new autoclaved water. At 24 hpi, larvae were fed larval food (ARTEMAC, Aquafauna Bio-Marine, Hawthorne, CA, USA). Water change, rinsing, and feeding were repeated daily following infection. Additionally, 100 µL of water from each experimental beaker was plated directly onto LB Agar with 100 µg/mL streptomycin and 100 µg/mL X-gal daily to confirm continued *V. cholerae* infection of the C6706 group and the sterility of uninfected group. At 9 dpf, individual larvae were placed in flat bottom 24-well plates (diameter 1.65-inch wells) (Falcon, MA, USA) to acclimate for 24 h. At 10 dpf, or 5 days post-infection (dpi), all 24-well plates were closed with a lid and sealed with parafilm around the edges, and light-dark or single-tap stimulus trials were performed.

### 2.3. Behavioral Assays

All behavioral assays were performed as previously described [49]. Briefly, the larvae in 24-well plates sealed with parafilm were placed in a DanioVision Observation Chamber (Noldus Information Technology, Wageningen, The Netherlands) and tracked using the EthoVision XT13 software and a Basler Gen1 Camera (Basler acA1300-60, Ahrensburg, Germany). Camera resolution was set at 1280 × 960 and the frame rate was set at 25. The DanioVision Temperature Control Unit maintained the larvae at a constant temperature of 28.0 ± 0.5 °C via a steady flow of water to the chamber. All behavioral trials were performed from 1–4 PM. Both behavioral assays were repeated in triplicate.

### 2.4. Light/Dark Trial

Larvae acclimated in the Noldus in darkness for 12 min. After acclimation, recording began and lasted for 24 min. A uniform light was emitted at 10,500 lux from below the stage. During all 4 alternating cycles of 3 min light and 3 min dark, distance moved (cm) and velocity (cm/s) were tracked (uninfected n = 25, C6706 n = 60). Average distance and average velocity were calculated in Excel from 30 s time bins and analyzed on GraphPad Prism 7.0, v4.02 using the Mann–Whitney test with *p* < 0.05 as cutoff for significance.

### 2.5. Single-Tap Trial

Larvae acclimated in the Noldus in darkness for 12 min. After acclimation, recording began and lasted for 2 min. Larvae were exposed to 1 min of no stimulation, followed by a single-tap at the highest intensity setting, and ending with 1 min of no stimulation. Distance moved (cm) was tracked (uninfected n = 63, C6706 n = 51). Average distance was calculated in Excel from 1 s time bins and analyzed on GraphPad using the Mann–Whitney test with *p* < 0.05 as cutoff for significance.

### 2.6. Zebrafish Euthanization and Homogenization

All larvae were euthanized in a lethal dose of MS-222 (300 mg/L Tris buffer, pH 7.0) for 30 min and homogenized with a pellet pestle (Fisher Scientific, Pittsburgh, PA, USA). Before infection, at 5 dpf, 15 larvae were homogenized together in 100 µL of 1× PBS. At 10 dpf, 5 uninfected larvae and 5 C6706-infected larvae were homogenized together in 100 µL of 1× PBS.

### 2.7. DNA Isolation and Sequencing

DNA from 100 µL of larval zebrafish’s combined homogenate was isolated using the DNEasy Powersoil Pro kit (Qiagen, Germantown, MD, USA) per the manufacturer’s instructions. Two extractions using only sterile 1× PBS and no gut samples were also processed as kit controls. The V4 region of the 16s rRNA gene was targeted and amplified using 515F and 806R primers. These primers have yielded successful results in previous zebrafish microbiome studies, and the V4 region has demonstrated high reproducibility [44,50]. Samples were submitted to Michigan State University for Illumina MiSeq Sequencing using previously established methods [51,52].

All raw data were processed with R package ‘dada2tools’, available at https://github.com/jp589/dada2tools (accessed on 29 July 2024), to efficiently correct Illumina amplicon errors without generating operational taxonomic units. Instead, amplicon sequence variants (ASVs) are derived based on 100% sequence similarity. Modifications to an online MiSeq protocol (https://benjjneb.github.io/dada2/tutorial.html, accessed on 29 July 2024) included allowing truncation lengths of 245 bp and 210 bp and a maximum number of errors of 2 bp for forward reads and 7 bp for reverse reads. Sequences were then classified into taxa using the silva_nr99_v138.1_train_set database with a minimum bootstrap value of 80% [53]. Sequences classified as mitochondrial, chloroplast, or not classified at phylum level were removed. Based on the bacterial profiles of two blank DNA extraction kit samples, 1 ASV, an unclassified *Corynebacterium*, was further removed from the dataset (https://github.com/jp589/dada2tools, accessed on 29 July 2024).

Sixty-five ASVs were detected in the larval gut microbiome. The samples of the gut microbiome of larvae at time zero yielded far fewer 16S rRNA gene sequencing reads (6772) than those of either uninfected control (252,894) or C6706 (181,224) larvae at five days post-infection. Thus, relative abundance data were used for descriptive comparisons among these three groups of samples.

## 3. Results

### 3.1. Light/Dark Trials

**Figure 1:**
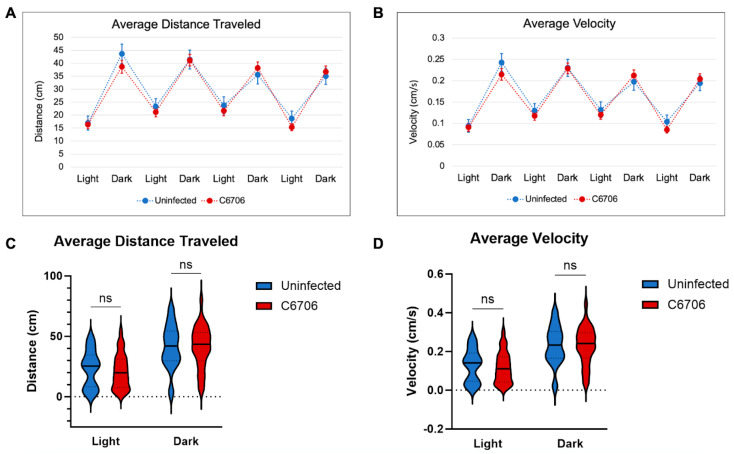
Light/Dark Trial Results. (**A**) Average distance traveled during 4 alternating periods of 3 min light and 3 min dark. Uninfected control in blue (n = 25) and C6706-infected in red (n = 60). (**B**) Average velocity during 4 alternating periods of 3 min light and 3 min dark. Uninfected control in blue (n = 25) and C6706-infected in red (n = 60). Error bars represent standard error of mean. (**C**) Violin plot of average distance traveled during the second cycle of light and dark. (**D**) Violin plot of average velocity during second cycle of light and dark. “ns” indicates no significance.

From the light/dark assay between uninfected larvae and C6706-colonized larvae, there was no significant difference in the average distance travelled nor average velocity of either condition (Figure 1A,B). Data distribution during the second cycle establishes the non-significant difference between the uninfected larvae and C6706-colonized larvae (Figure 1C,D). This trend continued throughout all four cycles.

### 3.2. Single-Tap Trials

**Figure 2:**
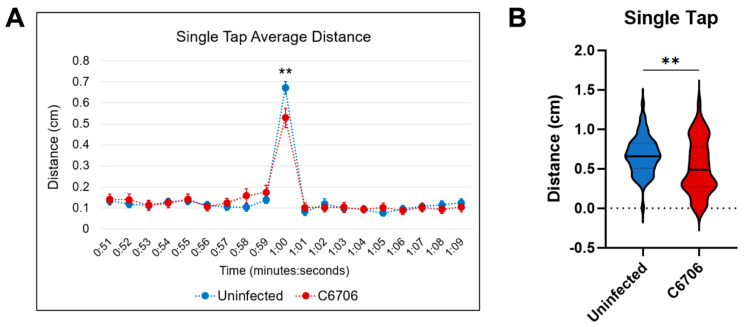
Single-tap trial results. (**A**) Uninfected control in blue (n = 63) and C6706-infected in red (n = 51). ** *p* = 0.0092. Student’s *t*-test performed for statistical significance. Error bars represent standard error of mean. (**B**) Violin plot of average distance traveled during single tap. “**” indicates *p* < 0.01.

From the single-tap assay, C6706-colonized larvae presented a significantly lower average distance traveled during the 1 s immediately after the single-tap when compared to the uninfected larvae (Figure 2A). The data distribution between the two groups was confirmed to be significant (Figure 2B).

### 3.3. Larval Gut Microbiome

**Figure 3:**
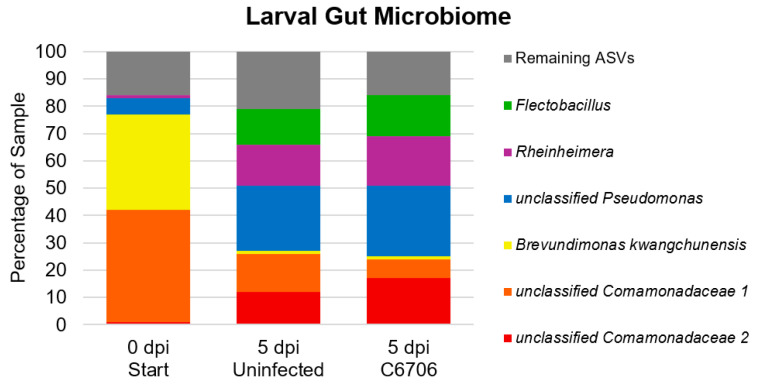
Stacked bar graph of most abundant ASVs identified in larval gut microbiome samples. “Remaining ASVs” refers to all ASVs that constitute less than 5% of the sample.

The two most abundant bacterial ASVs in the guts of larvae at time zero were mainly Proteobacteria, more specifically, an unclassified *Comamonadaceae* (41%) and *Brevundimonas kwangchunensis* (35%). At 5 dpi, the same unclassified *Comamonadaceae* ASV constituted 14% and 7% of the gut microbiomes of uninfected larvae and C6706-infected larvae, respectively. *B. kwangchunensis* constituted less than 1% of both larval groups’ microbiomes at this same timepoint. Only one other ASV, an unclassified *Pseudomonas*, constituted more than 5% of the gut microbiome of larvae at time zero. This ASV was the most prominent one among the gut microbiomes of both uninfected (24%) and C6706 (26%) larvae at 5 days post-infection. Notably, these two larval groups shared each of their top 5 ASVs, each constituting at least 5% of their gut microbiome profiles. The taxa of the remaining four ASVs were *Rheinheimera coerulea*, unclassified *Flectobacillus*, and two unclassified *Comamonadaceae* (Figure 3).

## 4. Discussion

The larval zebrafish brain has approximately 100,000 neurons [54]. Here, identifying a dampened motor response in C6706-colonized larvae to an acoustic stimulus, but not to visual stimuli, suggests that the gut microbiome has crosstalk with different regions and neurons of the larval brain. An open-source Zbrain atlas of the larval zebrafish brain has been established, opening the door for neuronal activity mapping [54]. Acoustic stimuli were found to activate the ears and lateral line, which directly connects to the octavolateralis nucleus (ON), as well as particularly strong neuron activations in the torus semicircularis, thalamus, cerebellum, and remaining hindbrain [54,55,56,57]. Visual stimulus in the form of a 10 s light flash activated the retinal projections and diencephalic areas of the larval brain [54]. In relation to our data, this could indicate a more direct pathway between the gut microbiome and the larval brain regions associated with a response to an acoustic stimulus. More specifically, a *V. cholerae* El Tor C6706-colonized gut microbiome is either indirectly or directly weakening crosstalk due to the presence of C6706 or absence of resident gut microbiota. Larval locomotion studies are translational in that activation of reticulospinal neurons in the brain stem and the vestibulospinal tract are conserved in vertebrates [58]. Structures of the larval zebrafish brain have evolutionarily conserved homologous functions to other vertebrates’ [59,60].

Our study aimed to identify any behavioral response from two separate stimuli. Screening for other larval behaviors may yield results highlighting other regions of the brain affected by a gut microbiome shift. This includes multi-tap assays to measure habituation, circadian rhythm assays, and prey-capture to measure decision-making [58,61,62,63,64]. Whole-brain imaging would be another direction towards larval neurology during *V. cholerae* colonization. Behavioral studies are also possible for adult zebrafish colonized with *V. cholerae*. While adult zebrafish exhibit much more complex, continuous behaviors, on a broader scale, translational relevance is equally complex [65]. For example, adult zebrafish social phenotypes were parallel to social interaction cues observed in humans, such as head direction and physical distance [66]. The link between *V. cholerae* colonization and behavior in zebrafish could mirror what is naturally occurring in the environment, where pathogenic strains of *V. cholerae* and zebrafish potentially interact. The link between a cholera infection of zebrafish and their resulting behavior could also demonstrate some advantage towards long-term *V. cholerae* El Tor gut colonization and the strain’s persistence in the aquatic environment.

Our study included characterizing the larval zebrafish gut microbiome to identify and validate any perturbation to abundance potentially caused by *V. cholerae* colonization. Comparison of our larval gut microbiome findings were relatively in line with similar previously established studies. The Comamonadaceae family, *Rheinheimera coerulea*, and Flectobacillus species, all of which were identified in our larval gut samples, have been isolated from freshwater environments [67,68,69]. *Pseudomonas*, which increased in abundance in the larval gut microbiome by 5 dpi, has been established as a part of the zebrafish core gut microbiome [46]. Stephens et al. have shown that unclassified Comamonadaceae took up 97.5% of all larval intestines, although this family was not as abundant in our samples [42]. *Brevundimonas kwangchunensis* is the one ASV of our top ASVs identified that has not been thoroughly described in literature, although the *Brevundimonas* genus has been found in soil and water samples [70,71]. It is interesting that *Vibrio* was not one of the most abundant ASVs from the C6706-infected group, though plating of 100 µL of two undiluted larval homogenates did yield X-gal blue CFUs indicative of *V. cholerae*, albeit at low counts of 4 and 11 CFUs. This low yield could potentially be explained by the small size of a single larval intestine. It could be possible that the specific group of larval homogenates submitted for sequencing were poorly infected and colonized with *V. cholerae* in comparison to other larvae. However, individual variations are statistically less significant in the behavioral study group colonized with *V. cholerae* El Tor C6706 (n = 111) compared to the gut microbiome collection group (n = 5). The gut microbiome abundances and number of ASVs significantly changing from 5 dpf to 10 dpf prove that gut microbiome diversity can quickly shift at this early developmental stage [42]. Additionally, perhaps plating homogenates on LB without streptomycin would provide confirmation of other, more abundant bacterial species’ growth. Since these behavioral analyses were the first to consider *V. cholerae*, future trials can include more larvae for sampling to provide a more comprehensive model of the gut microbiome.

In terms of V. cholerae colonization, while V. cholerae El Tor C6706 was tested, it is possible that classical and non-O1 biotype strains induce different behavioral responses. Future studies could determine if the interactions between the larval gut and brain play a role specific to the more persistent colonization of V. cholerae El Tor strains.

## Data Availability

Raw data is available from the corresponding author on request.
